# Hearing Loss in Alzheimer’s Disease Is Associated with Altered Serum Lipidomic Biomarker Profiles

**DOI:** 10.3390/cells9122556

**Published:** 2020-11-28

**Authors:** Daniel A. Llano, Lina K. Issa, Priya Devanarayan, Viswanath Devanarayan, Alzheimer’s Disease Neuroimaging Initiative (ADNI)

**Affiliations:** 1 Molecular and Cellular Biology, University of Illinois at Urbana-Champaign, Urbana, IL 61801, USA; lissa2@illinois.edu; 2 Carle Neuroscience Institute, Urbana, IL 61801, USA; 3 Department of Biology and Schreyer Honors College, Pennsylvania State University, University Park, PA 16802, USA; pxd5189@psu.edu; 4 GlaxoSmithKline, Collegeville, PA 19426 USA; devan@uic.edu; 5 Department of Mathematics, Statistics and Computer Science, University of Illinois at Chicago, Chicago, IL 60607, USA

**Keywords:** Alzheimer, mild cognitive impairment, hearing loss, presbycusis, phosphatidylcholine, lipidomic

## Abstract

Recent data have found that aging-related hearing loss (ARHL) is associated with the development of Alzheimer’s Disease (AD). However, the nature of the relationship between these two disorders is not clear. There are multiple potential factors that link ARHL and AD, and previous investigators have speculated that shared metabolic dysregulation may underlie the propensity to develop both disorders. Here, we investigate the distribution of serum lipidomic biomarkers in AD subjects with or without hearing loss in a publicly available dataset. Serum levels of 349 known lipids from 16 lipid classes were measured in 185 AD patients. Using previously defined co-regulated sets of lipids, both age- and sex-adjusted, we found that lipid sets enriched in phosphatidylcholine and phosphatidylethanolamine showed a strong inverse association with hearing loss. Examination of biochemical classes confirmed these relationships and revealed that serum phosphatidylcholine levels were significantly lower in AD subjects with hearing loss. A similar relationship was not found in normal subjects. These data suggest that a synergistic relationship may exist between AD, hearing loss and metabolic biomarkers, such that in the context of a pathological state such as AD, alterations in serum metabolic profiles are associated with hearing loss. These data also point to a potential role for phosphatidylcholine, a molecule with antioxidant properties, in the underlying pathophysiology of ARHL in the context of AD, which has implications for our understanding and potential treatment of both disorders.

## 1. Introduction

Aging-related hearing loss (ARHL) and Alzheimer’s Disease (AD) are common disabling disorders in the elderly. Over the age of 65, approximately 10% of individuals develop AD, while approximately 40% develop ARHL [1,2]. Both disorders are rising in prevalence as the population ages, and an estimated 83 million individuals will be over the age of 65 in the U.S. by the year 2050 [3]. Recent data have revealed an association between AD and ARHL, such that the likelihood of developing cognitive impairment, and ultimately AD, is increased in individuals with ARHL [4,5,6,7,8,9,10,11]. This relationship holds true even when adjusting for age, sex and multiple other potentially confounding variables, such as comorbid illness. A causal association has not been identified, though multiple mechanisms by which hearing loss may lead to AD have been proposed (reviewed in [12]).

ARHL and AD do share several potential biological substrates. Both are associated with metabolic stress and diminished mitochondrial function [13,14]. For example, in the cochlea, aging-related mitochondrial dysfunction may lead to chronic inflammation, resulting in the induction of apoptosis [15,16,17,18,19]. Lipid dysregulation may also play a role in the development of hearing loss [20]. ARHL is also associated with more traditional markers of AD, such as increases in cerebrospinal fluid (CSF) tau and diminished hippocampal and entorhinal cortical volume [21]. Recently, it has been suggested that AD may be associated with widespread dysregulation of lipid metabolism [22] and plasma lipid profiles have been shown to correlate with multiple AD-related biomarkers [23]. Further, lipid dysregulation in AD may lead to lipid peroxidation as well as dysregulation of brain inflammatory mediators, which are both associated with amyloid beta deposition [24,25,26]. It is therefore possible that an underlying process of metabolic dysregulation, including altered lipid homeostasis, may account for the relationship between AD and ARHL.

Lipids are a major component of biological membranes and integral to neuronal function. Body lipids are derived from three sources: our diet, adipose tissue storage and the liver’s synthetic capacity. Fats ingested in the diet enter the gastrointestinal tract, are digested by pancreatic lipases in the small intestine and are then moved across the intestinal mucosa. Lipids are then packaged along with cholesterol into chylomicrons which allow for nonpolar substances to move within the aqueous environment of our lymphatic and circulatory systems. These fats are then oxidized through β-oxidation for energy production or re-esterized for storage in adipose tissue. Alternatively, lipids in the small intestine can be distributed to the liver through portal circulation or to adipose tissue. Lipids derived from endogenous synthesis in the liver are packaged into very low-density lipoproteins that are transported to tissue or stored in adipose tissue. Fat stores in adipose tissue are mobilized for energy production by the action of hormone-sensitive lipase as needed. Lipids also form a key component of biological membranes and many have antioxidant properties. As such, disruptions in lipid metabolism are likely to cause widespread dysfunction in metabolically vulnerable tissues.

Given the potential roles for lipid dysregulation in the development of both AD and ARHL reviewed above, and recently discovered associations between serum lipid profiles and AD pathological biomarkers [23], we hypothesized that serum lipids may be disrupted in AD subjects with hearing loss. Therefore, in the current study, we examined the distribution of serum lipids in subjects with AD, with and without hearing loss, using a publicly available dataset (Alzheimer’s Disease Neuroimaging Initiative, ADNI).

## 2. Methods

### 2.1. Database

Data used in the preparation of this article were obtained from the Alzheimer’s Disease Neuroimaging Initiative (ADNI) database (adni.loni.usc.edu). The ADNI was launched in 2003 as a public–private partnership, led by principal investigator Michael W. Weiner, MD. The primary goal of the ADNI has been to test whether serial magnetic resonance imaging (MRI), positron emission tomography (PET), other biological markers and clinical and neuropsychological assessment can be combined to measure the progression of mild cognitive impairment (MCI) and early AD. For up-to-date information, see www.adni-info.org. This study was registered under ClinicalTrials.gov Identifier: NCT00106899. The study was conducted across multiple clinical sites and was approved by the Institutional Review Boards of all of the participating institutions. Informed written consent was obtained from all participants at each site. The following individual ethics boards approved the study: Albany Medical College Institutional Review Board, Boston University Medical Campus Institutional Review Board (BU IRB), Butler Hospital Institutional Review Board, Cleveland Clinic Institutional Review Board, Columbia University Institutional Review Board, Dartmouth-Hitchcock Medical Center Committee for the Protection of Human Subjects, Duke University Health System Institutional Review Board, Emory University Institutional Review Board, Georgetown University Institutional Review Board, Human Investigation Committee Yale University School of Medicine, Human Subjects Committee, University of Kansas Medical Center, Indiana University Institutional Review Board, Research Compliance Administration, Institutional Review Board of Baylor College of Medicine, Institutional Review Board of the Mount Sinai School of Medicine, Johns Hopkins University School of Medicine Institutional Review Boards, Lifespan—Rhode Island Hospital Institutional Review Board, Mayo Clinic Institutional Review Board, Nathan Kline Institute Rockland Psychiatric Center Institutional Review Board (NKI RPC IRB), New York University Langone Medical Center School of Medicine, Institutional Review Board Human Research Program, Northwestern University Institutional Review Board Office, Office of the Washington University School of Medicine IRB (OWUMC IRB), Oregon Health and Science University Institutional Review Board, Partners Human Research Committee, Research Ethics Board Jewish General Hospital, Research Ethics Board Sunnybrook Health Sciences Centre, Roper St. Francis Institutional Review Board, Rush University Medical Center Institutional Review Board, Stanford University, Administrative Panel on Human Subjects in Medical Research, The Ohio State University Institutional Review Board, The University of Texas Southwestern Medical Center Institutional Review Board, UCLA Office of the Human Research Protection Program Institutional Review Board, UCSD Human Research Protections Program, University Hospitals Case Medical Center Institutional Review Board, University of Alabama at Birmingham Institutional Review Board, University of British Columbia, Clinical Research Ethics Board (CREB), University of California Davis Office of Research IRB Administration, University of California Irvine Office of Research Institutional Review Board (IRB), University of California San Francisco Committee on Human Research (CHR), University of Iowa Institutional Review Board, University of Kentucky Office of Research Integrity, University of Michigan Medical School Institutional Review Board (IRBMED), University of Pennsylvania Institutional Review Board, University of Pittsburgh Institutional Review Board, University of Rochester Research Subjects Review Board (RSRB), University of South Florida Division of Research Integrity & Compliance, University of Southern California Health Science Campus Institutional Review Board, University of Western Ontario Research Ethics Board for Health Sciences Research Involving Human Subjects (HSREB), University of Wisconsin Health Sciences Institutional Review Board, Wake Forest University Institutional Review Board, Weill Cornell Medical College Institutional Review Board, Western Institutional Review Board and Western University Health Sciences Research Ethics Board. Data used for the analyses presented here were accessed on June 25, 2020.

### 2.2. Lipid Analysis

Details of lipid extraction and measurement as well as quality control measures have been previously described [27]. In brief, fasting serum samples were obtained from subjects during the baseline visit. Lipids were extracted using organic solvents. Serum extracts were then analyzed using liquid chromatography with mass spectrometry. After quality control measures, data were available from a total of 349 known lipids from 16 classes (see Table 1 for a list of lipid classes). The lipid subclasses in the ADNI serum lipidomics dataset used in this study include acylcarnitine, fatty acid, cholesteryl ester, lysophosphatidylcholine, lysophosphatidylethanolamine, phosphatidylcholine, phosphatidylethanolamine, phosphatidylinositol, plasmalogen phosphatidylcholine, plasmalogen phosphatidylethanolamine, ceramide, glucosylceramide, sphingomyelin, diacylglycerol and triacylglycerol (see Table 1 for a list of lipid classes).

### 2.3. Clinical Diagnosis and Hearing Loss Assessment

AD was diagnosed using NINCDS/ADRDA criteria for probable AD [28]. MCI patients had a memory complaint, an abnormal score on the Logical Memory II subscale from the Wechsler Memory Scale, a Mini-Mental Status Exam score between 24–30 and a Clinical Dementia Rating scale score of 0.5. Normal subjects did not have a memory complaint, had a normal score on the Logical Memory II subscale and had a Clinical Dementia Rating scale score of zero. Hearing was not systematically measured in the ADNI database. Similar to a previous report [21], we used subjective hearing loss complaints found in the following datasheets: ADSXLIST.csv, BLSCHECK.csv, INITHEALTH.csv, MEDHIST.csv, NEUROEXM.csv, PHYSICAL.csv, RECBLLOG.csv, RECMHIST.csv. We used the search terms “hear”, “auditory”, “ear”, “deaf”, “presbycusis” and “HOH (hard of hearing)” and eliminated those reports that were clearly not related to aging-related hearing loss (e.g., skin cancer on ear, earwax, etc.), as well as entries that referred to tinnitus without mention of hearing loss and eliminated duplicates. These search terms are identical to those used by Xu et al. (2019) and were selected prior to the data being seen. Subjects with a hearing complaint are labeled in this study as “hearing loss” or HL. Other subjects are listed as “non-hearing loss” or NHL, notwithstanding the fact that hearing was not objectively measured (see below).

### 2.4. Statistical Methods

The effect of each individual lipid species on hearing loss in AD subjects was assessed via analysis of covariance (ANCOVA) after adjusting for gender and age as covariates, and log transforming the lipid expression values. Samples with an absolute value of studentized residuals from this model exceeding 3 were identified as outliers and excluded from further analysis. The summary measures reported from this analysis include the area under the receiver operating characteristic curve (ROC AUC), covariate-adjusted significance (*p*-value) and false discovery rate [29].

The effect of each of the 16 known lipid classes and 28 empirically derived lipid sets (Barupal et al., 2019) on hearing loss in AD subjects was assessed via “lipid set analysis” (LSA). See Supplementary Table S1 for a list of the lipids in each of the 28 sets. This LSA of the lipid classes and lipid sets was based on the maxmean statistic of the gene set analysis algorithm [30], which was applied on the residuals from the above ANCOVA model on the individual lipid species to adjust for the effects of age and gender. Individual subject-level standardized composite scores were determined for each lipid class and each lipid set from this algorithm. These scores were then used to assess the effect of each of the lipid classes and lipid sets on hearing loss in AD subjects. The results were summarized in terms of ROC AUC, covariate-adjusted significance (*p*-value) and false discovery rate (*q*-value). Lipid sets with *q*-value < 0.05 were considered as statistically significant. The corresponding lipid classes and individual lipid species with Bonferroni-adjusted *p*-value < 0.05 were highlighted and studied further in terms of their potential connections to hearing loss in AD subjects.

## 3. Results

### 3.1. Demographics

Data were obtained from 185 subjects with AD. Of the 185, 40 (21.6%) reported hearing loss (HL). HL subjects were not significantly different in age than NHL subjects (HL: 77.2 ± 5.8 years (SD), NHL: 74.8 ± 7.7 years (SD), *p* > 0.05). HL subjects were more likely to be men than control subjects (NHL = 47% men, HL = 68% men, *p* < 0.05, chi-square). HL and NHL subjects did not differ significantly in average ADAS13 scores (HL: 30.4 ± 8.0 (SD), NHL: 28.8 ± 7.6 (SD), *p* > 0.05), body mass index (HL: 26.0 ± 4.1 kg/m^2^ (SD), NHL: 25.3 ± 3.8 kg/m^2^ (SD), *p* > 0.05) or use of prescription lipid-lowering drugs (e.g., statins, gemfibrozil, etc., HL: 55%, NHL: 52.4%, *p* = 0.77, chi-squared test, see Table 2).

### 3.2. Lipidomic Biomarker Sets That Separate HL from NHL Subjects

Levels of 349 lipids were measured across 16 classes. Because the levels of many of the lipids are strongly correlated due to co-regulation, and because of the high potential for false discovery when comparing the levels of all 349 lipids, we attempted to reduce the data by grouping the lipids. A previous report measured correlations between all of the serum lipid biomarkers, and using a dynamic clustering algorithm known as dynamicTreeCut, determined that 28 co-regulated sets of lipids were present [23]. They also found that many of these lipid sets were associated with either AD diagnosis or AD biomarkers. Although most of the sets were homogeneous (or near-homogeneous) clusters of single lipid types, others comprised a mixture of lipids (see supplementary Table S1 for a list of lipids in each class).

Given the robust performance of these clusters to signal changes in AD biomarkers, we asked whether these same clusters were also associated with the presence of HL. The *p*- and *q*-values for the 28 groups of lipids are shown in Table 3. We found that two sets of lipids correlated with the presence of hearing loss: set 23 and set 4, both with *p*- and *q*-values below 0.05, with set 23 producing the best performance. We therefore focused on the lipids found in these two sets for subsequent analyses of lipid class and individual lipids.

### 3.3. Lipid Classes and Individual Lipids That Separate HL from NHL Subjects

**Figure 1:**
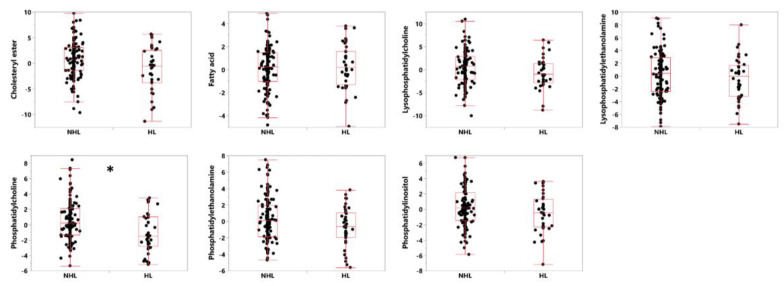
Box plots showing the median, first and third quartiles of the distributions, demonstrating differences in levels of lipids in the seven classes of lipids identified as parts of sets 4 and 23 from [23], distinguishing between HL and NHL in subjects with Alzheimer’s disease. Shown are standardized values (centered by mean and divided by standard deviation), after adjusting for age and gender as covariates. * Bonferroni-corrected *p*-value of < 0.05.

Using the biomarker sets to narrow our hypotheses about which lipids exhibit signal changes in hearing, we attempted to determine which lipid classes were most significantly associated with HL. Within the two significant sets identified above (*q* < 0.05), 25 lipids in seven classes were identified, with only the phosphatidylcholine class surviving correction for multiple comparisons (uncorrected *p*-value = 0.0057, Bonferroni corrected to 0.04). See Figure 1 for boxplots of the seven biomarker classes comparing HL and NHL subjects. See Table 4 for a list of lipid classes found in sets 4 and 23 and their associated capacity to separate HL from NHL subjects.

Among the 25 lipids in the two significant lipid sets identified above, the most commonly appearing lipid class was phosphatidylcholine (14/25 lipids or 56%), which is significantly greater than the proportion of all tested lipids that were in the phosphatidylcholine class (82/349 lipids or 23.4%, *p* < 0.05, chi-squared test). See Table 5 for a list of individual lipids in sets 4 and 23 and their associated capacity to separate HL from NHL subjects. Both of these analyses point to phosphatidylcholine levels as the main factor distinguishing between HL and NHL subjects.

### 3.4. Analysis of Non-AD Subjects and Apolipoprotein E (APOE)

Similar analyses were done in subjects with MCI (*n* = 225, 64 with HL) and control subjects without memory loss (*n* = 373, 104 with HL). None of the lipid sets were found to differentiate HL from NHL subjects in either control or MCI cohorts (see Table 6). Interaction of disease diagnosis (AD, MCI, control subjects) and hearing loss status (HL, NHL) with respect to specific lipid classes was formally assessed within the framework of a two-way ANOVA. Post hoc evaluation of this interaction effect from this model revealed that phosphatidylcholine was significantly differentiated between HL vs. NHL only in the AD subjects (*p* < 0.05), but not in the MCI and control subjects. Subjects across all groups (control, MCI and AD) were also separated based on genotype (having at least one copy of APOE4 or none), and no association was found between genotype and likelihood of HL.

## 4. Discussion

In the current study, 349 serum biomarkers were measured in 185 subjects with AD. Using previously identified co-regulated sets of biomarkers [23], we found two sets of lipids that were strongly associated with the presence of HL. Within these sets, the most common class of lipids was phosphatidylcholine, and as a class and as individual biomarkers, phosphatidylcholines were found to be significantly diminished in individuals with HL. Similar analyses in non-AD subjects (control and MCI) did not reveal significant associations between lipidomic biomarkers and HL

### 4.1. Weaknesses in the Study 

Hearing loss in this study was assessed in a non-systematic way—via subjective reports obtained from the subjects. Using the National Health and Nutrition Examination Survey (NHANES), which captured both objective hearing loss (using pure tone audiograms) and subjective hearing loss, previous data have established concordance values between subjective and objective hearing loss ranging from 65–77% depending on demographic factors [31]. Older subjects, such as the ones in this study, tended to underestimate their degree of hearing loss. These data suggest that some subjects with HL may have inappropriately been placed in the NHL category, and vice versa, but with a greater likelihood of missing HL subjects. Although there are several publicly available databases that have measured hearing loss objectively (e.g., the Baltimore Longitudinal Study of Aging or National Health and Nutrition Examination Survey), these did not systematically measure an extensive panel of lipid biomarkers. Conversely, despite the richness of biomarker data available in the ADNI, hearing was not systematically measured. Thus, additional future work in subjects with objectively-measured hearing loss will be required to confirm the associations reported here.

In addition, it is not possible to extrapolate the current findings to a therapeutic intervention. As an observational study, the current work cannot be used to support the idea that supplementation of phosphatidylcholine can protect against ARHL in subjects with AD. It is possible that phosphatidylcholine levels and ARHL are related by a third, unmeasured, factor. Only a prospective, randomized and blinded trial can determine whether phosphatidylcholine can improve ARHL.

### 4.2. Phosphatidylcholine, Alzheimer’s Disease and Hearing Loss

Phosphatidylcholine is one of the major phospholipids and a fundamental constituent of cell membranes and may activate enzymatic antioxidants situated in the cell membrane. There is also evidence for disrupted phosphatidylcholine metabolism in AD. For example, the enzymes that break down phosphatidylcholine (phospholipase D and phospholipase A2) are altered in AD [32,33]. In addition, low plasma levels of phosphatidylcholine docosahexaenoic acid have been associated with the development of AD [34] as well as thinning of the prefrontal cortex [35]. With respect to ARHL, phosphatidylcholine’s protective role in hearing loss was suggested by work from Seidman et al., who observed that lecithin (a polyunsaturated phosphatidylcholine) can protect against aging-related hearing loss in rats [36]. In this study, the investigators observed higher mitochondrial membrane potentials in the lecithin-treated group, suggesting preserved mitochondrial function. Lecithin treatment also diminished the occurrence mtDNA4834 deletion (common aging-related mitochondrial deletion) in the brain and cochlear tissue of the treated group. These data point to a role of phosphatidylcholine in protecting cochlear mitochondrial function. In addition, the antioxidants activated by phosphatidylcholine may protect the cell membrane from damage by reactive oxygen species [37] that arise during aging-related cochlear hypoperfusion, which can lead to cochlear degeneration [38,39]. These data all suggest that phosphatidylcholine levels may be depleted in AD and ARHL.

### 4.3. Origins of Measured Lipids

The lipids measured in this study were extracted from blood samples, which brings about the question of the origins of these lipids. Dietary fats are absorbed into the portal system to the liver. In the liver, fatty acids are incorporated into lipoprotein particles which are then released into the bloodstream. Additionally, adipocytes can release stored fatty acids into the blood as lipid levels in the blood decrease. Evidence also suggests that some fatty acids can be synthesized in the brain, but essential fatty acids still have to be transported across the blood–brain barrier [40]. Additional studies done on adult rats to study the rate of polyunsaturated fatty acid incorporation from plasma into the brain further suggests that this is a dynamic process with active daily turnover [41]. The exact mechanism behind how fats enter the brain is still unclear. One study performed on cholesterol homeostasis and hearing loss indicates that since the blood–brain barrier prevents the uptake of this lipoprotein from circulation, brain cholesterol is synthesized in astrocytes; further, excess cholesterol is metabolized into 24 (S)- hydroxycholesterol before secretion from the blood–brain barrier to the liver [42]. Thus, measured lipids in this study are likely derived from a variety of sources.

## 5. Conclusions

In the current study, we observed that in the context of AD, lower serum levels of phosphatidylcholine were associated with ARHL. The fact that this association was found in AD subjects, but not in non-AD subjects, suggests that there is an interaction between the presence of AD and the relationship between phosphatidylcholine and ARHL. Given that AD is associated with diminished brain mitochondrial function and increased levels of lipid peroxidation, it is possible that individuals with AD may not have the metabolic reserve to withstand additional metabolic stressors, such as declining levels of antioxidant molecules such as phosphatidylcholine. These data also suggest that normalizing phosphatidylcholine levels in AD subjects, but not in non-AD subjects, may have a role in the treatment or prevention of ARHL. Future studies will need to be done to investigate the potential therapeutic role of phosphatidylcholine in this context.

## Figures and Tables

**Table 1:** Listing of lipid classes in the current study.

Lipid Classes	Lipid Count
Acylcarnitine	9
Ceramide	19
Cholesterol	1
Cholesteryl ester	8
Diacylglycerol	13
Fatty acid	29
Galactoylceramide	1
Glucosylceramide	6
Lactosylceramide	1
Lysophosphatidylcholine	22
Lysophosphatidylethanolamine	4
Phosphatidylcholine	82
Phosphatidylethanolamine	25
Phosphatidylinositol	11
Sphingomyelin	34
Triacylglycerol	84

**Table 2:** Demographic variables. * *p* < 0.05. NHL = no hearing loss. HL = hearing loss.

		NHL	HL
*n* (# of AD subjects)		145	40
Gender * (*n*)	F	77	13
	M	68	27
Age in years (Mean +/− SD)		74.8 (7.7)	77.2 (5.8)
BMI in kg/m^2^ (Mean +/− SD)		25.32 (3.8)	26 (4.1)
Use of lipid-lowering drugs (*n*)	No	69	18
	Yes	76	22
ADAS13 (Mean +/− SD)		28.6 (7.6)	30.4 (8)

**Table 3:** Table of lipid sets derived from Barupal et al. (2019) and their performance in distinguishing HL from NHL in Alzheimer’s disease (AD) subjects. ROC AUC = receiver operating characteristic area under the curve. FDR-BH = Benjamini-Hochberg False Discovery Rate

Lipid Set	Median (NHL)	Median (HL)	ROC AUC	*p*-Value (unadj.)	*q*-Value (FDR-BH)
Set.23	0.54	−2.1	0.66	0.0006	0.0175
Set.4	0.16	−1.06	0.64	0.0032	0.0447
Set.6	−0.05	0.35	0.62	0.0148	0.1332
Set.25	1	−1.3	0.62	0.019	0.1332
Set.3	0.37	−0.74	0.6	0.0297	0.1664
Set.16	−0.16	0.27	0.59	0.0529	0.2171
Set.10	0.26	−1.18	0.6	0.0543	0.2171
Set.14	0.51	−1.16	0.6	0.0705	0.2467
Set.11	0.55	−1.21	0.61	0.0802	0.2495
Set.27	0.6	−1.61	0.58	0.1025	0.2871
Set.19	0.2	−0.35	0.58	0.1238	0.3014
Set.7	0.06	−0.74	0.58	0.1292	0.3014
Set.8	0.31	−0.26	0.57	0.1694	0.3467
Set.28	0.4	−0.12	0.56	0.1788	0.3467
Set.15	0.71	−0.31	0.56	0.1857	0.3467
Set.20	0.22	−0.45	0.56	0.2013	0.3523
Set.13	−0.67	−1.3	0.56	0.2554	0.4206
Set.17	0.34	0.2	0.47	0.2836	0.4387
Set.24	−0.4	−1.43	0.56	0.3135	0.4387
Set.2	−0.2	0.25	0.47	0.3153	0.4387
Set.9	0.54	−0.33	0.55	0.329	0.4387
Set.1	−0.3	−1.07	0.55	0.3879	0.4937
Set.26	0.02	0.58	0.48	0.4248	0.5171
Set.22	−0.18	−0.35	0.53	0.4606	0.5359
Set.18	−0.1	−0.68	0.54	0.4785	0.5359
Set.5	0.54	−0.55	0.53	0.6265	0.6747
Set.21	−0.21	0.32	0.5	0.8784	0.9109
Set.12	0.09	0.07	0.51	0.9827	0.9827

**Table 4:** Table of lipid classes derived from sets 4 and 23 from Barupal et al. (2019) and their performance in distinguishing HL from NHL subjects. Unadjusted *p*-values that survive the Bonferroni correction (<0.05) are noted with *.

Lipid Class	Median (NHL)	Median (HL)	ROC AUC	*p*-Value (Unadjusted)
Phosphatidylcholine	0.25	−1.48	0.63	0.0057 *
Phosphatidylethanolamine	0.13	−0.6	0.59	0.0216
Cholesteryl ester	0.58	−0.53	0.62	0.0239
Phosphatidylinositol	0.16	−0.45	0.58	0.1142
Lysophosphatidylcholine	0.48	−0.93	0.58	0.1255
Lysophosphatidylethanolamine	0.35	−0.02	0.55	0.3185
Fatty acid	0.12	0.22	0.54	0.3344

**Table 5:** Table of lipids derived from sets 4 and 23 from Barupal et al., 2019 and their performance in distinguishing HL from NHL subjects.

Lipid ID	Lipid Class	Lipid Set	Median (NHL)	Median (HL)	Fold Change (HL/NHL)	*p*-Value
UCD.Lipid.162	Phosphatidylcholine	Set-23	63,671	52,397	0.82	0.0003
UCD.Lipid.163	Phosphatidylcholine	Set-23	29,367	24,419.5	0.83	0.0006
UCD.Lipid.148	Phosphatidylcholine	Set-4	5,056,746	4,380,450.5	0.87	0.0010
UCD.Lipid.161	Phosphatidylcholine	Set-23	46,893	39,148.5	0.83	0.0014
UCD.Lipid.164	Phosphatidylcholine	Set-23	20,525	16,734.5	0.82	0.0033
UCD.Lipid.17	Cholesteryl ester	Set-4	254,617	198,944.5	0.78	0.0055
UCD.Lipid.150	Phosphatidylcholine	Set-4	58,706	49,712	0.85	0.0069
UCD.Lipid.406	Phosphatidylcholine	Set-4	130,587	116,420	0.89	0.0079
UCD.Lipid.128	Lysophosphatidylcholine	Set-4	45,190	37,318.5	0.83	0.0130
UCD.Lipid.451	Phosphatidylethanolamine	Set-23	5726.33	4968	0.87	0.0149
UCD.Lipid.143	Phosphatidylcholine	Set-4	23,780	19,794	0.83	0.0150
UCD.Lipid.409	Phosphatidylcholine	Set-4	69,527	57,894	0.83	0.0163
UCD.Lipid.149	Phosphatidylcholine	Set-4	35,516.5	27,777	0.78	0.0166
UCD.Lipid.462	Phosphatidylinositol	Set-4	9408	8256	0.88	0.0183
UCD.Lipid.447	Phosphatidylethanolamine	Set-23	12,761	11,301.5	0.89	0.0197
UCD.Lipid.450	Phosphatidylethanolamine	Set-23	12,513.79	10,888.5	0.87	0.0217
UCD.Lipid.410	Phosphatidylcholine	Set-4	7910	7552	0.95	0.0310
UCD.Lipid.145	Phosphatidylcholine	Set-4	22,891	19,311	0.84	0.0329
UCD.Lipid.126	Lysophosphatidylcholine	Set-4	14,172.5	12,585	0.89	0.0428
UCD.Lipid.399	Phosphatidylcholine	Set-4	98,283	79,011	0.80	0.0661
UCD.Lipid.16	Cholesteryl ester	Set-4	179,126	125,757	0.70	0.0846
UCD.Lipid.442	Phosphatidylethanolamine	Set-4	1817.5	1492	0.82	0.1016
UCD.Lipid.381	Lysophosphatidylethanolamine	Set-4	5577.5	5206	0.93	0.2087
UCD.Lipid.517	Fatty acid	Set-4	111,966	100,323	0.90	0.5032
UCD.Lipid.513	Fatty acid	Set-4	22,307	21,665	0.97	0.7021

**Table 6:** Table of lipid sets derived from Barupal et al. (2019) and their performance in distinguishing HL from NHL in mild cognitive impairment (MCI) and NL subjects. ROC AUC = receiver operating characteristic area under the curve. Top ten sets shown for each group of subjects.

**MCI Subjects**					
**Lipid Set**	**Median (HN)**	**Median (HL)**	**ROC AUC**	***p*-Value (unadj.)**	***q*-Value (FDR-BH)**
Set.13	−0.91	−0.42	0.54	0.0538	0.9348
Set.22	0.03	0.82	0.53	0.0775	0.9348
Set.14	−0.26	0.17	0.53	0.1122	0.9348
Set.25	0.19	−0.08	0.49	0.1824	0.9468
Set.16	−0.22	0.26	0.56	0.23	0.9468
Set.18	−1.34	−1.33	0.51	0.3203	0.9468
Set.27	0.31	−0.15	0.55	0.363	0.9468
Set.8	0.04	0.32	0.51	0.4406	0.9468
Set.17	0.03	−0.1	0.5	0.4798	0.9468
Set.24	−1.51	−0.39	0.51	0.5114	0.9468
**Subjects without Memory Loss or Complaint**					
**Lipid Set**	**Median (HN)**	**Median (HL)**	**ROC AUC**	***p*-Value (unadj.)**	***q*-Value (FDR-BH)**
Set.22	0.4	1.7	0.59	0.0092	0.2293
Set.17	0.48	−0.84	0.6	0.0502	0.4158
Set.14	0.37	0.54	0.54	0.0632	0.4158
Set.13	0.14	1.03	0.57	0.0818	0.4158
Set.3	−0.1	−0.07	0.53	0.0832	0.4158
Set.7	−0.33	−0.18	0.54	0.1062	0.4163
Set.8	−0.37	0.74	0.57	0.1166	0.4163
Set.11	−0.49	−0.24	0.54	0.1438	0.4495
Set.20	−0.14	−0.17	0.55	0.2283	0.5529
Set.23	1.25	1.24	0.48	0.2393	0.5529

## Supplementary Materials

The following are available online at https://www.mdpi.com/2073-4409/9/12/2556/s1.
