# Supplementary material for: Hearing Loss in Alzheimer’s Disease Is Associated with Altered Serum Lipidomic Biomarker Profiles
Source: Cells. 2020 Nov 28;9(12):2556. doi: 10.3390/cells9122556 (PMC7760745; doi:10.3390/cells9122556)
Supplement: Supplementary file 1 [file cells-09-02556-s001.pdf]

Supplemental Table 1: Listing of lipids in all empirically-derived sets from Barupal et al. 2019

Lipid Set	Lipid Class	Lipid ID	Network Label	FullName	AcylChainInformation	Adduct	RT	MZ
Set-1	Triacylglycerol	UCD.Lipid.173	TG 42:0	TG (42:0)	TG (14:0_14:0_14:0)	[M+NH4]+	9.43	740.6763
Set-1	Triacylglycerol	UCD.Lipid.174	TG 40:0	TG (40:0)	TG(12:0_12:0_16:0)	[M+NH4]+	9.1	712.6419
Set-1	Triacylglycerol	UCD.Lipid.177	TG 42:2	TG (42:2)	TG(12:0_12:0_18:2)	[M+NH4]+	8.467	736.6446
Set-1	Triacylglycerol	UCD.Lipid.179	TG 44:0	TG (44:0)	TG(14:0_14:0_16:0)	[M+NH4]+	10.13	768.7037
Set-1	Triacylglycerol	UCD.Lipid.180	TG 44:1	TG (44:1)	TG(12:0_14:0_18:1)	[M+NH4]+	9.455	766.6919
Set-1	Triacylglycerol	UCD.Lipid.181	TG 44:2	TG (44:2)		[M+Na]+	9.033	769.6316
Set-1	Triacylglycerol	UCD.Lipid.182	TG 46:0	TG (46:0)	TG(14:0_16:0_16:0)	[M+NH4]+	10.38	796.7389
Set-1	Triacylglycerol	UCD.Lipid.183	TG 46:1	TG (46:1)	TG(12:0_16:0_18:1)	[M+NH4]+	9.944	794.7232
Set-1	Triacylglycerol	UCD.Lipid.184	TG 46:2	TG (46:2)	TG(12:0_16:1_18:1)	[M+Na]+	9.512	797.663
Set-1	Triacylglycerol	UCD.Lipid.185	TG 46:3	TG (46:3)	TG(12:0_16:1_18:2)	[M+Na]+	9.065	795.6473
Set-1	Triacylglycerol	UCD.Lipid.186	TG 46:4 A	TG (46:4) A		[M+Na]+	8.8	793.6322
Set-1	Triacylglycerol	UCD.Lipid.187	TG 48:0	TG (48:0)	TG(16:0_16:0_16:0)	[M+NH4]+	10.81	824.7702
Set-1	Triacylglycerol	UCD.Lipid.188	TG 48:1	TG (48:1)	TG(14:0_16:0_18:1)	[M+NH4]+	10.4	822.7545
Set-1	Triacylglycerol	UCD.Lipid.189	TG 48:2	TG (48:2)	TG(14:0_16:0_18:2)	[M+NH4]+	9.993	820.7389
Set-1	Triacylglycerol	UCD.Lipid.192	TG 49:0	TG (49:0)	TG(16:0_16:0_17:0)	[M+NH4]+	10.94	838.7858
Set-1	Triacylglycerol	UCD.Lipid.193	TG 49:1	TG (49:1)	TG(15:0_16:0_18:1)	[M+NH4]+	10.6	836.7702
Set-1	Triacylglycerol	UCD.Lipid.194	TG 49:2	TG (49:2)	TG(15:0_16:0_18:2)	[M+NH4]+	10.18	834.7545
Set-1	Triacylglycerol	UCD.Lipid.196	TG 50:0	TG (50:0)	TG(16:0_16:0_18:0)	[M+NH4]+	11.21	852.8015
Set-1	Triacylglycerol	UCD.Lipid.197	TG 50:1	TG (50:1)	TG(16:0_16:0_18:1)	[M+NH4]+	10.82	850.7858
Set-1	Triacylglycerol	UCD.Lipid.198	TG 50:2	TG (50:2)	TG(16:0_16:1_18:1)	[M+NH4]+	10.42	848.7702
Set-1	Triacylglycerol	UCD.Lipid.202	TG 50:6	TG (50:6)	TG(14:0_16:1_20:5)	[M+Na]+	9.18	845.6599
Set-1	Triacylglycerol	UCD.Lipid.203	TG 51:1	TG (51:1)	TG(16:0_17:0_18:1)	[M+NH4]+	10.96	864.8015
Set-1	Triacylglycerol	UCD.Lipid.208	TG 52:0	TG (52:0)	TG(16:0_18:0_18:0)	[M+NH4]+	11.58	880.8328
Set-1	Triacylglycerol	UCD.Lipid.209	TG 52:1	TG (52:1)	TG(16:0_18:0_18:1)	[M+NH4]+	11.22	878.8171
Set-1	Triacylglycerol	UCD.Lipid.215	TG 53:1	TG (53:1)	TG(17:0_18:0_18:1)	[M+NH4]+	11.35	892.8328
Set-1	Triacylglycerol	UCD.Lipid.220	TG 54:0	TG (54:0)	TG(18:0_18:0_18:0)	[M+NH4]+	11.92	908.8576
Set-1	Triacylglycerol	UCD.Lipid.221	TG 54:1	TG (54:1)	TG(18:0_18:0_18:1)	[M+NH4]+	11.58	906.8484
Set-1	Triacylglycerol	UCD.Lipid.229	TG 56:2	TG (56:2)	TG(18:1_18:1_20:0)	[M+NH4]+	11.58	932.8641
Set-2	Cholesteroyl ester	UCD.Lipid.10	CE 16:1	CE (16:1)	16:1 Cholesteryl ester	[M+Na]+	10.33	645.5581
Set-2	Lysophosphatidylcholine	UCD.Lipid.119	LPC 14:0	LPC (14:0)	LysoPC 14:0	[M+H]+	0.981	468.3089
Set-2	Phosphatidylcholine	UCD.Lipid.133	PC 28:0	PC (28:0)		[M+H]+	4.239	678.5065

Set -2	Phosphatidylcholine	UCD.Lipid.134	PC 30:0	PC (30:0)	PC(14:0_16:0)	[M+H]+	4.811	706.5378
Set -2	Phosphatidylcholine	UCD.Lipid.135	PC 30:1	PC (30:1)		[M+H]+	4.322	704.5225
Set -2	Phosphatidylcholine	UCD.Lipid.136	PC 31:0	PC (31:0)		[M+H]+	5.11	720.5538
Set -2	Phosphatidylcholine	UCD.Lipid.137	PC 31:1	PC (31:1)		[M+H]+	4.82	718.5345
Set -2	Phosphatidylcholine	UCD.Lipid.138	PC 32:3	PC (32:3)		[M+H]+	4.811	728.5222
Set -2	Phosphatidylcholine	UCD.Lipid.139	PC 33:0	PC (33:0)		[M+H]+	5.64	748.5851
Set -2	Phosphatidylcholine	UCD.Lipid.383	PC 32:1	PC (32:1)	PC(16:0_16:1)	[M+Ac–H]–	5.033	790.56
Set -2	Phosphatidylcholine	UCD.Lipid.384	PC 32:2	PC (32:2)	PC(14:0_18:2)	[M+Ac–H]–	4.567	788.54
Set -2	Phosphatidylcholine	UCD.Lipid.385	PC 33:1	PC (33:1)	PC(15:0_18:1)	[M+Ac–H]–	5.34	804.58
Set -2	Phosphatidylcholine	UCD.Lipid.388	PC 34:1	PC (34:1)	PC(16:0_18:1)	[M+Ac–H]–	5.648	818.59
Set -2	Phosphatidylcholine	UCD.Lipid.390	PC 34:3	PC (34:3)	PC(16:0_18:3)	[M+Ac–H]–	4.75	814.56
Set -2	Phosphatidylcholine	UCD.Lipid.391	PC 34:4	PC (34:4)	PC(14:0_20:4)	[M+Ac–H]–	4.492	812.54
Set -2	Phosphatidylcholine	UCD.Lipid.392	PC 35:1	PC (35:1)	PC(17:0_18:1)	[M+Ac–H]–	5.973	832.61
Set -2	Phosphatidylcholine	UCD.Lipid.395	PC 36:1	PC (36:1)	PC(18:0_18:1)	[M+Ac–H]–	6.314	846.62
Set -2	Phosphatidylethanolamine	UCD.Lipid.431	PE 36:1	PE (36:1)	PE(18:0_18:1)	[M–H]–	5.648	744.55
Set -2	Phosphatidylethanolamine	UCD.Lipid.437	PE 40:6	PE (40:6)	PE(18:0_22:6)	[M–H]–	4.908	790.54
Set -2	Phosphatidylinositol	UCD.Lipid.452	PI 32:1	PI (32:1)	PI(16:0_16:1)	[M–H]–	4.025	807.5
Set -2	Phosphatidylinositol	UCD.Lipid.458	PI 36:4	PI (36:4)	PI(16:0_20:4)	[M–H]–	4.092	857.52
Set -3	Sphingomyelin	UCD.Lipid.169	SM d30:1	SM (d30:1)	SM (d18:1_12:0)	[M+H]+	3.634	647.5123
Set -3	Ceramide	UCD.Lipid.257	Cer d32:1	Ceramide (d32:1)	Cer[NS](d16:1_16:0)	[M+Ac–H]–	5.091	568.49
Set -3	Ceramide	UCD.Lipid.258	Cer d33:1	Ceramide (d33:1)	Cer[NS](d17:1_16:0)	[M+Cl]–	5.415	558.47
Set -3	Ceramide	UCD.Lipid.260	Cer d34:1	Ceramide (d34:1)	Cer[NS](d18:1_16:0)	[M+Ac–H]–	5.748	596.53
Set -3	Ceramide	UCD.Lipid.261	Cer d34:2	Ceramide (d34:2)		[M+Ac–H]–	5.182	594.51
Set -3	Ceramide	UCD.Lipid.262	Cer d36:1	Ceramide (d36:1)	Cer[NS](d18:1_18:0)	[M+Cl]–	6.455	600.51
Set -3	Ceramide	UCD.Lipid.272	Cer d43:1	Ceramide (d43:1)	Cer[NS](d19:1_24:0)	[M+Cl]–	8.682	698.62
Set -3	Sphingomyelin	UCD.Lipid.463	SM d32:0	SM (d32:0)		[M+Ac–H]–	4.467	735.56
Set -3	Sphingomyelin	UCD.Lipid.464	SM d32:1	SM (d32:1)	SM(d18:1_14:0)	[M+Ac–H]–	4.267	733.55
Set -3	Sphingomyelin	UCD.Lipid.465	SM d32:2	SM (d32:2)		[M+Ac–H]–	3.818	731.53
Set -3	Sphingomyelin	UCD.Lipid.466	SM d33:1	SM (d33:1)	SM(d17:1_16:0)	[M+Ac–H]–	4.558	747.57
Set -3	Sphingomyelin	UCD.Lipid.469	SM d36:0	SM (d36:0)		[M+Ac–H]–	5.765	791.63
Set -3	Sphingomyelin	UCD.Lipid.470	SM d36:1	SM (d36:1)	SM(d18:1_18:0)	[M+Ac–H]–	5.523	789.61
Set -3	Sphingomyelin	UCD.Lipid.473	SM d37:1	SM (d37:1)		[M+Ac–H]–	5.873	803.63
Set -3	Sphingomyelin	UCD.Lipid.474	SM d38:0	SM (d38:0)	SM(d18:0_20:0)	[M+Ac–H]–	6.455	819.66
Set -3	Sphingomyelin	UCD.Lipid.475	SM d38:1	SM (d38:1)	SM(d18:1_20:0)	[M+Ac–H]–	6.214	817.64

Set -3	Sphingomyelin	UCD.Lipid.477	SM d39:1	SM (d39:1)	SM(d16:1_23:0)	[M+Ac–H]–	6.555	831.66
Set -3	Sphingomyelin	UCD.Lipid.490	SM d43:1	SM (d43:1)		[M+Ac–H]–	7.77	887.72
Set -3	Sphingomyelin	UCD.Lipid.491	SM d43:2	SM (d43:2)		[M+Ac–H]–	7.079	885.71
Set -4	Lysophosphatidylcholine	UCD.Lipid.126	LPC 20:5	LPC (20:5)	LysoPC 20:5	[M+H]+	0.964	542.3239
Set -4	Lysophosphatidylcholine	UCD.Lipid.128	LPC 22:6	LPC (22:6)	LysoPC 22:6	[M+H]+	1.155	568.3404
Set -4	Phosphatidylcholine	UCD.Lipid.143	PC 36:6	PC (36:6)		[M+H]+	4.231	778.5378
Set -4	Phosphatidylcholine	UCD.Lipid.145	PC 37:6	PC (37:6)		[M+H]+	4.496	792.5538
Set -4	Phosphatidylcholine	UCD.Lipid.148	PC 38:6 A	PC (38:6) A	PC(16:0_22:6	[M+H]+	4.778	806.5691
Set -4	Phosphatidylcholine	UCD.Lipid.149	PC 38:7	PC (38:7)		[M+H]+	4.314	804.5538
Set -4	Phosphatidylcholine	UCD.Lipid.150	PC 39:6	PC (39:6)		[M+H]+	5.027	820.5851
Set -4	Cholesteroyl ester	UCD.Lipid.16	CE 20:5	CE (20:5)	20:5 Cholesteryl ester	[M+Na]+	9.711	693.5581
Set -4	Cholesteroyl ester	UCD.Lipid.17	CE 22:6	CE (22:6)	22:6 Cholesteryl ester	[M+Na]+	9.894	719.5738
Set -4	Lysophosphatidylethanolamine	UCD.Lipid.381	LPE 22:6	LPE (22:6)	LysoPC 22:6	[M–H]–	1.263	524.28
Set -4	Phosphatidylcholine	UCD.Lipid.399	PC 36:5 B	PC (36:5) B	PC(16:1_20:4)	[M+Ac–H]–	4.683	838.56
Set -4	Phosphatidylcholine	UCD.Lipid.406	PC 38:6	PC (38:6)	PC(18:1_20:5)_PC(18:2_20:4)	[M+Ac–H]–	4.908	864.58
Set -4	Phosphatidylcholine	UCD.Lipid.409	PC 40:6 B	PC (40:6) B	PC(18:0_22:6)	[M+Ac–H]–	5.532	892.61
Set -4	Phosphatidylcholine	UCD.Lipid.410	PC 40:7	PC (40:7)	PC(18:1_22:6)	[M+Ac–H]–	4.983	890.59
Set -4	Phosphatidylethanolamine	UCD.Lipid.442	PE p-36:5	PE (p-36:5) or PE (o-36:6)	PE(P-16:0_20:5)	[M–H]–	5.099	720.5
Set -4	Phosphatidylinositol	UCD.Lipid.462	PI 40:6	PI (40:6)	PI(18:0_22:6)	[M–H]–	4.508	909.55
Set -4	Fatty acid	UCD.Lipid.513	FA 20:5	FA (20:5)	FA (20:5)	[M–H]–	1.53	301.22
Set -4	Fatty acid	UCD.Lipid.517	FA 22:6	FA (22:6)	FA (22:6)	[M–H]–	1.72	327.23
Set -5	Diacylglycerol	UCD.Lipid.104	DG 32:1	DG (32:1)	DG(16:0_16:1)	[M+Na]+	6.143	589.4812
Set -5	Diacylglycerol	UCD.Lipid.105	DG 34:1	DG (34:1)	DG(16:0_18:1)	[M+Na]+	6.892	617.5116
Set -5	Diacylglycerol	UCD.Lipid.106	DG 34:2	DG (34:2)	DG(16:0_18:2)	[M+Na]+	6.27	615.4965
Set -5	Diacylglycerol	UCD.Lipid.107	DG 34:3	DG (34:3)	DG(16:1_18:2)	[M+NH4]+	5.686	608.5246
Set -5	Diacylglycerol	UCD.Lipid.109	DG 36:2	DG (36:2)	DG(18:1_18:1)	[M+NH4]+	6.95	638.5718
Set -5	Diacylglycerol	UCD.Lipid.110	DG 36:3	DG (36:3)	DG(18:1_18:2)	[M+NH4]+	6.42	636.5562
Set -5	Triacylglycerol	UCD.Lipid.190	TG 48:3	TG (48:3)	TG(12:0_18:1_18:2)	[M+Na]+	9.546	823.6789
Set -5	Triacylglycerol	UCD.Lipid.191	TG 48:4	TG (48:4)	TG(12:0_18:2_18:2)	[M+Na]+	9.34	821.6597
Set -5	Triacylglycerol	UCD.Lipid.195	TG 49:3	TG (49:3)	TG(15:0_16:1_18:2)	[M+NH4]+	9.794	832.7389
Set -5	Triacylglycerol	UCD.Lipid.199	TG 50:3	TG (50:3)	TG(16:1_16:1_18:1)	[M+NH4]+	10.02	846.7545
Set -5	Triacylglycerol	UCD.Lipid.200	TG 50:4	TG (50:4)	TG(16:1_16:1_18:2)	[M+Na]+	9.629	849.6943
Set -5	Triacylglycerol	UCD.Lipid.201	TG 50:5	TG (50:5)	TG(14:0_18:2_18:3)	[M+Na]+	9.24	847.6786
Set -5	Triacylglycerol	UCD.Lipid.204	TG 51:2	TG (51:2)	TG(16:0_17:0_18:2)	[M+NH4]+	10.62	862.7858

Set-5	Triacylglycerol	UCD.Lipid.205	TG 51:3	TG (51:3)	TG(15:0_18:1_18:2)	[M+NH4]+	10.25	860.7702
Set-5	Triacylglycerol	UCD.Lipid.210	TG 52:2	TG (52:2)	TG(16:0_18:1_18:1)	[M+NH4]+	10.83	876.8015
Set-5	Triacylglycerol	UCD.Lipid.211	TG 52:3	TG (52:3)	TG(16:0_18:1_18:2)	[M+NH4]+	10.47	874.7858
Set-5	Triacylglycerol	UCD.Lipid.216	TG 53:2	TG (53:2)	TG(17:0_18:1_18:1)	[M+NH4]+	11.01	890.8171
Set-5	Triacylglycerol	UCD.Lipid.217	TG 53:3	TG (53:3)	TG(17:0_18:1_18:2)	[M+NH4]+	10.65	888.8015
Set-6	Diacylglycerol	UCD.Lipid.108	DG 36:1	DG (36:1)	DG(18:0_18:1)	[M+NH4]+	7.47	640.588
Set-6	Diacylglycerol	UCD.Lipid.113	DG 38:0	DG (38:0)		[M+NH4]+	7.94	670.6086
Set-6	Diacylglycerol	UCD.Lipid.114	DG 38:3	DG (38:3)	DG(20:1_18:2)	[M+Na]+	6.974	669.5653
Set-6	Diacylglycerol	UCD.Lipid.116	DG 38:6	DG (38:6)	DG(18:2_20:4)	[M+Na]+	5.732	663.4959
Set-6	Lysophosphatidylcholine	UCD.Lipid.127	LPC 22:4	LPC (22:4)	LysoPC 22:4	[M+H]+	1.661	572.3711
Set-6	Lysophosphatidylcholine	UCD.Lipid.129	LPC o-16:0	LPC (o-16:0)	LPC (o-16:0)	[M+H]+	1.752	482.3606
Set-6	Lysophosphatidylcholine	UCD.Lipid.130	LPC p-16:0	LPC (p-16:0) or LPC (o-16:1)	LPC (o-16:1)	[M+H]+	1.694	480.3448
Set-6	Lysophosphatidylcholine	UCD.Lipid.131	LPC p-18:0	LPC (p-18:0) or LPC (o-18:1)	LPC (o-18:1)	[M+H]+	1.909	508.3762
Set-6	Triacylglycerol	UCD.Lipid.175	TG 40:1	TG (40:1)		[M+NH4]+	8.384	710.6298
Set-6	Triacylglycerol	UCD.Lipid.178	TG 42:3	TG (42:3)		[M+NH4]+	8.24	734.6265
Set-6	Ceramide	UCD.Lipid.19	Cer d42:2	Ceramide (d42:2)	Cer[NS](d18:2_24:0)	[M+H]+	7.615	648.6289
Set-6	Triacylglycerol	UCD.Lipid.253	TG 60:6	TG (60:6)		[M+Na]+	11.27	985.8105
Set-6	Ceramide	UCD.Lipid.259	Cer d34:0	Ceramide (d34:0)	Cer[NDS](d18:0_16:0)	[M+Ac–H]–	5.998	598.54
Set-6	Phosphatidylethanolamine	UCD.Lipid.439	PE p-36:1	PE (p-36:1) or PE (o-36:2)	PE(P-18:0_18:1)	[M–H]–	6.838	728.56
Set-6	Fatty acid	UCD.Lipid.511	FA 20:3	FA (20:3)	FA (20:3)	[M–H]–	2.5	305.25
Set-6	Fatty acid	UCD.Lipid.512	FA 20:4	FA (20:4)	FA (20:4)	[M–H]–	1.93	303.23
Set-6	Fatty acid	UCD.Lipid.515	FA 22:1	FA (22:1)	FA (22:1)	[M–H]–	3.9	337.31
Set-6	Acylcarnitine	UCD.Lipid.9	AC C18:3	Acylcarnitine C18:3	Acylcarnitine 18:3	[M+H]+	1.13	422.3265
Set-7	Phosphatidylcholine	UCD.Lipid.142	PC 36:4 A	PC (36:4) A	PC(18:2_18:2)	[M+H]+	4.637	782.5694
Set-7	Glucosylceramide	UCD.Lipid.368	GlcCerd 24:1-2OH	GlcCer(d14:1(4E)/20:0(2OH))		[M+Cl]–	4.966	750.53
Set-7	Phosphatidylcholine	UCD.Lipid.382	PC 32:0	PC (32:0)	PC(16:0_16:0)	[M+Ac–H]–	5.573	792.58
Set-7	Phosphatidylcholine	UCD.Lipid.386	PC 33:2	PC (33:2)	PC(16:0_18:2)	[M+Ac–H]–	4.858	802.56
Set-7	Phosphatidylcholine	UCD.Lipid.387	PC 34:0	PC (34:0)	PC(16:0_18:0)	[M+Ac–H]–	6.239	820.61
Set-7	Phosphatidylcholine	UCD.Lipid.389	PC 34:2	PC (34:2)	PC(16:0_18:2)	[M+Ac–H]–	5.166	816.58
Set-7	Phosphatidylcholine	UCD.Lipid.393	PC 35:2	PC (35:2)	PC(17:0_18:2)	[M+Ac–H]–	5.48	830.59
Set-7	Phosphatidylcholine	UCD.Lipid.396	PC 36:2	PC (36:2)	PC(18:0_18:2)	[M+Ac–H]–	5.806	844.61
Set-7	Phosphatidylcholine	UCD.Lipid.400	PC 37:2	PC (37:2)	PC(19:0_18:2)	[M+Ac–H]–	6.14	858.62
Set-7	Phosphatidylethanolamine	UCD.Lipid.434	PE 38:2	PE (38:2)	PE(20:0_18:2)	[M–H]–	5.806	770.57

Set -7	Phosphatidylethanolamine	UCD.Lipid.443	PE p-38:2	PE (p-38:2) or PE (o-38:3)		[M–H]–	6.98	754.58
Set -7	Phosphatidylethanolamine	UCD.Lipid.448	PE p-40:4	PE (p-40:4) or PE (o-40:5)	PE(P-20:0_20:4)	[M–H]–	6.87	778.58
Set -7	Phosphatidylinositol	UCD.Lipid.453	PI 34:1	PI (34:1)	PI(16:0_18:1)	[M–H]–	4.483	835.53
Set -7	Phosphatidylinositol	UCD.Lipid.454	PI 34:2	PI (34:2)	PI(16:0_18:2)	[M–H]–	4.158	833.52
Set -7	Phosphatidylinositol	UCD.Lipid.455	PI 36:1	PI (36:1)	PI(18:0_18:1)	[M–H]–	5.198	863.57
Set -7	Phosphatidylinositol	UCD.Lipid.456	PI 36:2	PI (36:2)	PI(18:0_18:2)	[M–H]–	4.732	861.55
Set -7	Phosphatidylinositol	UCD.Lipid.457	PI 36:3	PI (36:3)	PI(16:0_20:3)	[M–H]–	4.225	859.53
Set -8	Lysophosphatidylcholine	UCD.Lipid.120	LPC 15:0	LPC (15:0)	LysoPC 15:0	[M+H]+	1.206	482.3241
Set -8	Lysophosphatidylcholine	UCD.Lipid.121	LPC 17:1	LPC (17:1)	LysoPC 17:1	[M+H]+	1.345	508.3401
Set -8	Lysophosphatidylcholine	UCD.Lipid.122	LPC 18:0	LPC (18:0)	LysoPC 18:0	[M+H]+	2.258	524.3708
Set -8	Lysophosphatidylcholine	UCD.Lipid.123	LPC 18:3	LPC (18:3)	LysoPC 18:3	[M+H]+	0.981	518.3241
Set -8	Lysophosphatidylcholine	UCD.Lipid.124	LPC 20:0	LPC (20:0)	LysoPC 20:0	[M+H]+	3.062	552.4021
Set -8	Lysophosphatidylcholine	UCD.Lipid.369	LPC 16:0	LPC (16:0)	LysoPC 16:0	[M+Ac–H]–	1.563	554.35
Set -8	Lysophosphatidylcholine	UCD.Lipid.370	LPC 16:1	LPC (16:1)	LysoPC 16:1	[M+Ac–H]–	1.18	552.33
Set -8	Lysophosphatidylcholine	UCD.Lipid.371	LPC 18:0 A	LPC (18:0) A	LysoPC 18:0	[M+Ac–H]–	2.145	582.38
Set -8	Lysophosphatidylcholine	UCD.Lipid.372	LPC 18:1	LPC (18:1)	LysoPC 18:1	[M+Ac–H]–	1.713	580.36
Set -8	Lysophosphatidylcholine	UCD.Lipid.373	LPC 18:2	LPC (18:2)	LysoPC 18:2	[M+Ac–H]–	1.313	578.35
Set -8	Lysophosphatidylcholine	UCD.Lipid.374	LPC 20:1	LPC (20:1)	LysoPC 20:1	[M+Ac–H]–	2.47	608.39
Set -8	Lysophosphatidylcholine	UCD.Lipid.375	LPC 20:2	LPC (20:2)	LysoPC 20:2	[M+Ac–H]–	1.896	606.38
Set -8	Lysophosphatidylcholine	UCD.Lipid.376	LPC 20:3	LPC (20:3)	LysoPC 20:3	[M+Ac–H]–	1.505	604.36
Set -8	Lysophosphatidylcholine	UCD.Lipid.377	LPC 22:5	LPC (22:5)	LysoPC 22:5	[M+Ac–H]–	1.388	628.36
Set -8	Lysophosphatidylethanolamine	UCD.Lipid.378	LPE 16:0	LPE (16:0)	LysoPE 16:0	[M–H]–	1.596	452.28
Set -8	Lysophosphatidylethanolamine	UCD.Lipid.379	LPE 18:2	LPE (18:2)	LysoPE 18:2	[M–H]–	1.347	476.28
Set -9	Phosphatidylcholine	UCD.Lipid.132	PC 27:0-CHO	PC (16:0/9:0(CHO))	PC (16:0_9:0(CHO))	[M+H]+	2.374	650.4391
Set -9	Triacylglycerol	UCD.Lipid.176	TG 42:1	TG (42:1)	TG(12:0_12:0_18:1)	[M+Na]+	8.967	743.6162
Set -9	Fatty acid	UCD.Lipid.493	FA 11:0	FA (11:0)	FA (11:0)	[M–H]–	0.82	185.15
Set -9	Fatty acid	UCD.Lipid.494	FA 12:0	FA (12:0)	FA (12:0)	[M–H]–	1.06	199.17
Set -9	Fatty acid	UCD.Lipid.495	FA 13:0	FA (13:0)	FA (13:0)	[M–H]–	1.3	213.19
Set -9	Fatty acid	UCD.Lipid.496	FA 14:0	FA (14:0)	FA (14:0)	[M–H]–	1.63	227.2
Set -9	Fatty acid	UCD.Lipid.498	FA 15:0	FA (15:0)	FA (15:0)	[M–H]–	2.02	241.22
Set -9	Fatty acid	UCD.Lipid.499	FA 15:1	FA (15:1)	FA (15:1)	[M–H]–	1.5	239.2
Set -9	Fatty acid	UCD.Lipid.500	FA 16:0	FA (16:0)	FA (16:0)	[M–H]–	2.47	255.23
Set -9	Fatty acid	UCD.Lipid.502	FA 17:0	FA (17:0)	FA (17:0)	[M–H]–	2.96	269.25
Set -9	Fatty acid	UCD.Lipid.504	FA 18:0	FA (18:0)	FA (18:0)	[M–H]–	3.26	283.26

Set -9	Fatty acid	UCD.Lipid.508	FA 20:0	FA (20:0)	FA (20:0)	[M–H]–	3.85	311.3
Set -9	Fatty acid	UCD.Lipid.514	FA 22:0	FA (22:0)	FA (22:0)	[M–H]–	4.5	339.33
Set -9	Fatty acid	UCD.Lipid.518	FA 24:0	FA (24:0)	FA (24:0)	[M–H]–	5.23	367.36
Set -9	Fatty acid	UCD.Lipid.520	FA 26:0	FA (26:0)	FA (26:0)	[M–H]–	6.02	395.39
Set -9	Fatty acid	UCD.Lipid.521	FA 28:0	FA (28:0)	FA (28:0)	[M–H]–	6.82	423.42
Set -10	Sphingomyelin	UCD.Lipid.171	SM d40:2	SM (d40:2)		[M+H]+	6.038	785.6531
Set -10	Phosphatidylinositol	UCD.Lipid.461	PI 38:5	PI (38:5)	PI(18:1_20:4)	[M–H]–	4.142	883.53
Set -10	Sphingomyelin	UCD.Lipid.468	SM d34:2	SM (d34:2)	SM(d18:2_16:0)	[M+Ac–H]–	4.359	759.57
Set -10	Sphingomyelin	UCD.Lipid.471	SM d36:2	SM (d36:2)		[M+Ac–H]–	4.974	787.6
Set -10	Sphingomyelin	UCD.Lipid.472	SM d36:3	SM (d36:3)		[M+Ac–H]–	4.508	785.58
Set -10	Sphingomyelin	UCD.Lipid.476	SM d38:2	SM (d38:2)		[M+Ac–H]–	5.623	815.63
Set -10	Sphingomyelin	UCD.Lipid.478	SM d39:2	SM (d39:2)		[M+Ac–H]–	5.988	829.64
Set -10	Sphingomyelin	UCD.Lipid.479	SM d40:0	SM (d40:0)		[M+Ac–H]–	7.138	847.69
Set -10	Sphingomyelin	UCD.Lipid.480	SM d40:1	SM (d40:1)	SM(d18:1_22:0)	[M+Ac–H]–	6.888	845.67
Set -10	Sphingomyelin	UCD.Lipid.481	SM d40:2 A	SM (d40:2) A		[M+Ac–H]–	6.205	843.66
Set -10	Sphingomyelin	UCD.Lipid.482	SM d40:2 B	SM (d40:2) B	SM(d18:2_22:0)	[M+Ac–H]–	6.306	843.66
Set -10	Sphingomyelin	UCD.Lipid.483	SM d40:3	SM (d40:3)		[M+Ac–H]–	5.64	841.64
Set -10	Sphingomyelin	UCD.Lipid.484	SM d41:1	SM (d41:1)	SM(d18:1_23:0)	[M+Ac–H]–	7.229	859.69
Set -10	Sphingomyelin	UCD.Lipid.485	SM d41:2	SM (d41:2)	SM(d18:2_23:0)	[M+Ac–H]–	6.65	857.67
Set -10	Sphingomyelin	UCD.Lipid.486	SM d42:0	SM (d42:0)		[M+Ac–H]–	7.794	875.72
Set -10	Sphingomyelin	UCD.Lipid.487	SM d42:1	SM (d42:1)	SM(d18:1_24:0)	[M+Ac–H]–	7.562	873.71
Set -11	Glactoylceramide	UCD.Lipid.117	LacCer d34:1	Gal-Gal-Cer(d18:1/16:0) or Lactosylceramide(d18:1/16:0)		[M+H]+	4.795	862.625
Set -11	Lactosylceramide	UCD.Lipid.118	LacCer d32:2	Lactosylceramide (d18:1/24:1(15Z))		[M+H]+	6.685	972.7346
Set -11	Cholesteroyl ester	UCD.Lipid.12	CE 18:2	CE (18:2)	18:2 Cholesteryl ester	[M+NH4]+	10.4	666.6184
Set -11	Phosphatidylcholine	UCD.Lipid.155	PC o-34:0	PC (o-34:0)		[M+H]+	6.478	748.6204
Set -11	Phosphatidylcholine	UCD.Lipid.156	PC p-32:1	PC (p-32:1) or PC (o-32:2)		[M+H]+	4.91	716.5626
Set -11	Phosphatidylcholine	UCD.Lipid.157	PC p-34:1	PC (p-34:1) or PC (o-34:2) B	PC(P-16:0_18:1)	[M+H]+	5.79	744.5908
Set -11	Phosphatidylcholine	UCD.Lipid.160	PC p-40:1	PC (p-40:1) or PC (o-40:2)		[M+H]+	6.637	828.6842
Set -11	Sphingomyelin	UCD.Lipid.170	SM d34:0	SM (d34:0)		[M+H]+	4.96	705.5905
Set -11	Glucosylceramide	UCD.Lipid.363	GlcCer d38:1	GlcCer (d38:1)		[M+Ac–H]–	6.489	814.64
Set -11	Glucosylceramide	UCD.Lipid.364	GlcCer d40:1	GlcCer (d40:1)	GlcCer[NS](d18:1_22:0)	[M+Ac–H]–	7.163	842.67
Set -11	Glucosylceramide	UCD.Lipid.365	GlcCer d41:1	GlcCer (d41:1)		[M+Ac–H]–	7.495	856.69
Set -11	Glucosylceramide	UCD.Lipid.366	GlcCer d42:1	GlcCer (d42:1)	GlcCer[NS](d18:1_24:0)	[M+Ac–H]–	7.82	870.7

Set -11	Glucosylceramide	UCD.Lipid.367	GlcCer d42:2	GlcCer (d42:2)	GlcCer[NS](d18:1_24:1)	[M+Ac–H]–	7.138	868.69
Set -11	Phosphatidylcholine	UCD.Lipid.412	PC o-32:0	PC (o-32:0)		[M+Ac–H]–	5.973	778.59
Set -11	Phosphatidylcholine	UCD.Lipid.414	PC p-34:0	PC (p-34:0) or PC (o-34:1)	PC(P-18:0_16:0)	[M+Ac–H]–	6.039	804.61
Set -11	Sphingomyelin	UCD.Lipid.467	SM d34:1	SM (d34:1)	SM(d18:1_16:0)	[M+Ac–H]–	4.866	761.58
Set -12	Diacylglycerol	UCD.Lipid.111	DG 36:4	DG (36:4)	DG(18:2_18:2)	[M+NH4]+	5.815	634.5404
Set -12	Diacylglycerol	UCD.Lipid.112	DG 36:5	DG (36:5)	DG(18:2_18:3)	[M+NH4]+	5.65	632.5248
Set -12	Triacylglycerol	UCD.Lipid.206	TG 51:4	TG (51:4)	TG(15:0_18:2_18:2)	[M+Na]+	9.877	863.7099
Set -12	Triacylglycerol	UCD.Lipid.207	TG 51:5	TG (51:5)	TG(15:0_18:2_18:3)	[M+NH4]+	9.77	856.7347
Set -12	Triacylglycerol	UCD.Lipid.212	TG 52:4	TG (52:4)	TG(16:1_18:1_18:2)	[M+NH4]+	10.1	872.7702
Set -12	Triacylglycerol	UCD.Lipid.213	TG 52:5	TG (52:5)	TG(16:0_18:2_18:3)	[M+NH4]+	9.728	870.7545
Set -12	Triacylglycerol	UCD.Lipid.214	TG 52:6	TG (52:6)	TG(16:1_18:2_18:3)	[M+Na]+	9.35	873.6943
Set -12	Triacylglycerol	UCD.Lipid.218	TG 53:4	TG (53:4)	TG(17:1_18:1_18:2)	[M+NH4]+	10.32	886.7858
Set -12	Triacylglycerol	UCD.Lipid.219	TG 53:5	TG (53:5)	TG(17:1_18:2_18:2)	[M+NH4]+	9.902	884.7702
Set -12	Triacylglycerol	UCD.Lipid.223	TG 54:3	TG (54:3)	TG(18:0_18:1_18:2)	[M+NH4]+	10.86	902.8171
Set -12	Triacylglycerol	UCD.Lipid.224	TG 54:4	TG (54:4)	TG(18:1_18:1_18:2)	[M+NH4]+	10.49	900.8015
Set -12	Triacylglycerol	UCD.Lipid.225	TG 54:5	TG (54:5)	TG(18:1_18:2_18:2)	[M+Na]+	10.12	903.7412
Set -12	Triacylglycerol	UCD.Lipid.226	TG 54:6	TG (54:6)	TG(18:1_18:2_18:3)	[M+Na]+	9.745	901.7256
Set -12	Triacylglycerol	UCD.Lipid.227	TG 54:8	TG (54:8)	TG(18:2_18:3_18:3)	[M+Na]+	8.94	897.6943
Set -13	Triacylglycerol	UCD.Lipid.228	TG 56:1	TG (56:1)	TG(18:0_18:1_20:0)	[M+NH4]+	11.85	934.8797
Set -13	Triacylglycerol	UCD.Lipid.237	TG 57:1	TG (57:1)		[M+NH4]+	12	948.8863
Set -13	Triacylglycerol	UCD.Lipid.238	TG 57:2	TG (57:2)		[M+NH4]+	11.88	946.8718
Set -13	Triacylglycerol	UCD.Lipid.239	TG 58:1	TG (58:1)	TG(18:0_18:1_22:0)	[M+NH4]+	11.99	962.911
Set -13	Triacylglycerol	UCD.Lipid.241	TG 58:2	TG (58:2)	TG(18:1_18:1_22:0)	[M+NH4]+	11.85	960.8954
Set -13	Triacylglycerol	UCD.Lipid.242	TG 58:3	TG (58:3)	TG(18:1_20:1_20:1)	[M+NH4]+	11.6	958.8797
Set -13	Triacylglycerol	UCD.Lipid.247	TG 59:2	TG (59:2)		[M+NH4]+	12	974.9031
Set -13	Triacylglycerol	UCD.Lipid.248	TG 59:3	TG (59:3)		[M+NH4]+	11.89	972.8871
Set -13	Triacylglycerol	UCD.Lipid.250	TG 60:2	TG (60:2)	TG(18:1_20:1_22:0)	[M+NH4]+	11.99	988.9267
Set -13	Triacylglycerol	UCD.Lipid.251	TG 60:3	TG (60:3)	TG(18:1_20:1_22:1)	[M+NH4]+	11.95	986.9034
Set -13	Triacylglycerol	UCD.Lipid.252	TG 60:4	TG (60:4)	TG(18:1_20:2_22:1)	[M+NH4]+	11.78	984.8877
Set -13	Triacylglycerol	UCD.Lipid.254	TG 62:3	TG (62:3)		[M+NH4]+	12.04	1014.934
Set -13	Triacylglycerol	UCD.Lipid.255	TG 62:4	TG (62:4)		[M+NH4]+	11.94	1012.918
Set -13	Triacylglycerol	UCD.Lipid.256	TG 64:4	TG (64:4)		[M+NH4]+	12.04	1040.949
Set -14	Ceramide	UCD.Lipid.18	Cer d41:1	Ceramide (d18:1/23:0)	Cer[NS](d18:1_23:0)	[M+H]+	7.963	636.6289
Set -14	Ceramide	UCD.Lipid.263	Cer d38:1	Ceramide (d38:1)	Cer[NS](d18:1_20:0)	[M+Cl]–	7.163	628.54

Set -14	Ceramide	UCD.Lipid.264	Cer d39:1	Ceramide (d39:1)	Cer[NS](d18:1_22:0)	[M+Cl]–	7.512	642.56
Set -14	Ceramide	UCD.Lipid.265	Cer d40:0	Ceramide (d40:0)	Cer[NDS](d18:0_22:0)	[M+Ac–H]–	8.061	682.63
Set -14	Ceramide	UCD.Lipid.266	Cer d40:1	Ceramide (d40:1)	Cer[NS](d18:1_22:0)	[M+Cl]–	7.836	656.58
Set -14	Ceramide	UCD.Lipid.267	Cer d40:2	Ceramide (d40:2)	Cer[NS](d18:2_22:0)	[M+Cl]–	7.185	654.56
Set -14	Ceramide	UCD.Lipid.268	Cer d41:1	Ceramide (d41:1)	Cer[NS](d18:1_23:0)	[M+Cl]–	8.161	670.59
Set -14	Ceramide	UCD.Lipid.269	Cer d42:0	Ceramide (d42:0)	Cer[NDS](d18:0_24:0)	[M+Cl]–	8.685	686.62
Set -14	Ceramide	UCD.Lipid.270	Cer d42:1	Ceramide (d42:1)	Cer[NS](d18:1_24:0)	[M+Ac–H]–	8.469	708.65
Set -14	Ceramide	UCD.Lipid.271	Cer d42:2 A	Ceramide (d42:2) A	Cer[NS](d18:1_24:1)	[M+Cl]–	NA	NA
Set -14	Ceramide	UCD.Lipid.273	Cer d44:1	Ceramide (d44:1)		[M+Cl]–	9.068	712.64
Set -15	Phosphatidylcholine	UCD.Lipid.159	PC p-38:4	PC (p-38:4) or PC (o-38:5) B	PC(P-18:0_20:4)	[M+H]+	5.823	794.6054
Set -15	Phosphatidylethanolamine	UCD.Lipid.167	PE 38:4	PE (38:4)	PE(18:0_20:4)	[M+H]+	5.723	768.556
Set -15	Phosphatidylcholine	UCD.Lipid.413	PC p-32:0	PC (p-32:0) or PC (o-32:1)	PC(P-16:0_16:0)	[M+Ac–H]–	5.881	776.58
Set -15	Phosphatidylcholine	UCD.Lipid.415	PC p-34:1	PC (p-34:1) or PC (o-34:2) A	PC(P-16:0_18:1)	[M+Ac–H]–	5.54	802.6
Set -15	Phosphatidylcholine	UCD.Lipid.416	PC p-34:2	PC (p-34:2) or PC (o-34:3)	PC(P-16:0_18:2)	[M+Ac–H]–	5.44	800.58
Set -15	Phosphatidylcholine	UCD.Lipid.417	PC p-36:1	PC (p-36:1) or PC (o-36:2)	PC(P-18:0_18:1)	[M+Ac–H]–	6.2	830.63
Set -15	Phosphatidylcholine	UCD.Lipid.418	PC p-36:2	PC (p-36:2) or PC (o-36:3)	PC(P-18:0_18:2)	[M+Ac–H]–	5.59	828.61
Set -15	Phosphatidylcholine	UCD.Lipid.419	PC p-36:3	PC (p-36:3) or PC (o-36:4)	PC(P-16:0_20:3)	[M+Ac–H]–	5.44	826.6
Set -15	Phosphatidylcholine	UCD.Lipid.420	PC p-36:4	PC (p-36:4) or PC (o-36:5)	PC(P-16:0_20:4)	[M+Ac–H]–	5.332	824.58
Set -15	Phosphatidylcholine	UCD.Lipid.422	PC p-38:4	PC (p-38:4) or PC (o-38:5) A	PC(P-18:0_20:4)	[M+Ac–H]–	5.482	852.61
Set -15	Phosphatidylcholine	UCD.Lipid.423	PC p-38:5	PC (p-38:5) or PC (o-38:6)	PC(P-18:0_20:5)	[M+Ac–H]–	5.257	850.6
Set -16	Acylcarnitine	UCD.Lipid.1	AC C8:0	Acylcarnitine C8:0	Acylcarnitine 8:0	[M+H]+	0.55	288.2169
Set -16	Acylcarnitine	UCD.Lipid.2	AC C8:1	Acylcarnitine C8:1	Acylcarnitine 8:1	[M+H]+	0.52	286.2013
Set -16	Cholesterol	UCD.Lipid.20	Cholesterol	Cholesterol		[M–H2O+H]+	4.861	369.3516
Set -16	Acylcarnitine	UCD.Lipid.3	AC C10:1	Acylcarnitine C10:1	Acylcarnitine 10:1	[M+H]+	0.59	314.2326
Set -16	Acylcarnitine	UCD.Lipid.4	AC C12:0	Acylcarnitine C12:0	Acylcarnitine 12:0	[M+H]+	0.74	344.2801
Set -16	Acylcarnitine	UCD.Lipid.5	AC C16:0	Acylcarnitine C16:0	Acylcarnitine 16:0	[M+H]+	1.586	400.342
Set -16	Fatty acid	UCD.Lipid.519	FA 24:1	FA (24:1)	FA (24:1)	[M–H]–	4.52	365.34
Set -16	Acylcarnitine	UCD.Lipid.6	AC C18:0	Acylcarnitine C18:0	Acylcarnitine 18:0	[M+H]+	2.34	428.3727
Set -16	Acylcarnitine	UCD.Lipid.7	AC C18:1	Acylcarnitine C18:1	Acylcarnitine 18:1	[M+H]+	1.743	426.3578
Set -16	Acylcarnitine	UCD.Lipid.8	AC C18:2	Acylcarnitine C18:2	Acylcarnitine 18:2	[M+H]+	1.329	424.3425
Set -17	Fatty acid	UCD.Lipid.497	FA 14:1	FA (14:1)	FA (14:1)	[M–H]–	1.2	225.19
Set -17	Fatty acid	UCD.Lipid.501	FA 16:1	FA (16:1)	FA (16:1)	[M–H]–	1.82	253.22
Set -17	Fatty acid	UCD.Lipid.503	FA 17:1	FA (17:1)	FA (17:1)	[M–H]–	2.22	267.23

Set -17	Fatty acid	UCD.Lipi d.505	FA 18:1	FA (18:1)	FA (18:1)	[M–H]–	2.7	281.25
Set -17	Fatty acid	UCD.Lipi d.506	FA 18:2	FA (18:2)	FA (18:2)	[M–H]–	2.05	279.23
Set -17	Fatty acid	UCD.Lipi d.507	FA 18:3	FA (18:3)	FA (18:3)	[M–H]–	1.623	277.22
Set -17	Fatty acid	UCD.Lipi d.509	FA 20:1	FA (20:1)	FA (20:1)	[M–H]–	3.35	309.28
Set -17	Fatty acid	UCD.Lipi d.510	FA 20:2	FA (20:2)	FA (20:2)	[M–H]–	2.91	307.26
Set -17	Fatty acid	UCD.Lipi d.516	FA 22:2	FA (22:2)	FA (22:2)	[M–H]–	3.47	335.3
Set -18	Diacylglycerol	UCD.Lipi d.115	DG 38:5	DG (38:5)	DG(18:1_20:4)	[M+NH4]+	6.246	660.5562
Set -18	Triacylglycerol	UCD.Lipi d.222	TG 54:2	TG (54:2)	TG(18:0_18:1_18:1)	[M+NH4]+	11.22	904.8328
Set -18	Triacylglycerol	UCD.Lipi d.230	TG 56:3	TG (56:3)	TG(18:0_18:1_20:2)	[M+NH4]+	11.22	930.8484
Set -18	Triacylglycerol	UCD.Lipi d.231	TG 56:4	TG (56:4)	TG(18:0_18:1_20:3)	[M+NH4]+	10.9	928.8328
Set -18	Triacylglycerol	UCD.Lipi d.232	TG 56:5	TG (56:5)	TG(18:0_18:1_20:4)	[M+NH4]+	10.78	926.8171
Set -18	Triacylglycerol	UCD.Lipi d.233	TG 56:6	TG (56:6)	TG(18:1_18:1_20:4)	[M+NH4]+	10.34	924.8015
Set -18	Triacylglycerol	UCD.Lipi d.234	TG 56:7	TG (56:7)	TG(18:1_18:2_20:4)	[M+NH4]+	9.968	922.7858
Set -18	Triacylglycerol	UCD.Lipi d.243	TG 58:4	TG (58:4)	TG(18:1_18:2_22:1)	[M+NH4]+	11.25	956.8641
Set -18	Triacylglycerol	UCD.Lipi d.244	TG 58:6	TG (58:6)	TG(18:0_18:1_22:5)	[M+NH4]+	10.67	952.8328
Set -19	Phosphatidylcholine	UCD.Lipi d.147	PC 38:5 B	PC (38:5) B	PC(16:0_22:5)	[M+H]+	5.168	808.5848
Set -19	Phosphatidylcholine	UCD.Lipi d.151	PC 40:5 B	PC (40:5) B	PC(18:0_22:5)	[M+H]+	5.806	836.6164
Set -19	Phosphatidylcholine	UCD.Lipi d.152	PC 40:6 A	PC (40:6) A	PC(18:0_22:6)	[M+H]+	5.151	834.6007
Set -19	Phosphatidylcholine	UCD.Lipi d.153	PC 42:5	PC (42:5)		[M+H]+	6.072	864.645
Set -19	Phosphatidylcholine	UCD.Lipi d.154	PC 42:6	PC (42:6)		[M+H]+	5.773	862.6311
Set -19	Phosphatidylcholine	UCD.Lipi d.405	PC 38:5 A	PC (38:5) A	PC(18:1_20:4)	[M+Ac–H]–	5.107	866.59
Set -19	Phosphatidylcholine	UCD.Lipi d.408	PC 40:5 A	PC (40:5) A	PC(18:0_22:5)	[M+Ac–H]–	5.74	894.62
Set -19	Phosphatidylcholine	UCD.Lipi d.411	PC 40:8	PC (40:8)	PC(18:2_22:6)	[M+Ac–H]–	4.533	888.58
Set -19	Phosphatidylinositol	UCD.Lipi d.460	PI 38:4	PI (38:4)	PI(18:0_20:4)	[M–H]–	4.606	885.55
Set -20	Phosphatidylcholine	UCD.Lipi d.158	PC p-38:2	PC (p-38:2) or PC (o-38:3)		[M+H]+	6.212	798.6359
Set -20	Phosphatidylcholine	UCD.Lipi d.165	PC p-42:3	PC (p-42:3) or PC (o-42:4)		[M+H]+	7.224	852.6859
Set -20	Phosphatidylcholine	UCD.Lipi d.421	PC p-38:3	PC (p-38:3) or PC (o-38:4)	PC(P-18:0_20:3)	[M+Ac–H]–	6.098	854.63
Set -20	Phosphatidylcholine	UCD.Lipi d.424	PC p-40:3	PC (p-40:3) or PC (o-40:4)		[M+Ac–H]–	6.763	882.66
Set -20	Phosphatidylcholine	UCD.Lipi d.425	PC p-40:4	PC (p-40:4) or PC (o-40:5)	PC(P-20:0_20:4)	[M+Ac–H]–	6.106	880.64
Set -20	Phosphatidylcholine	UCD.Lipi d.426	PC p-42:4	PC (p-42:4) or PC (o-42:5)	PC(P-22:0_20:4)	[M+Ac–H]–	6.747	908.67
Set -20	Phosphatidylcholine	UCD.Lipi d.427	PC p-42:5	PC (p-42:5) or PC (o-42:6)		[M+Ac–H]–	6.188	906.66
Set -20	Phosphatidylcholine	UCD.Lipi d.428	PC p-44:4	PC (p-44:4) or PC (o-44:5)		[M+Ac–H]–	7.387	936.71
Set -21	Lysophosphatidylcholine	UCD.Lipi d.125	LPC 20:4	LPC (20:4)	LysoPC 20:4	[M+H]+	1.205	544.3403
Set -21	Phosphatidylcholine	UCD.Lipi d.146	PC 38:4 B	PC (38:4) B		[M+H]+	6.146	810.6004

Set -21	Lysophosphatidylethanolamine	UCD.Lipid.380	LPE 20:4	LPE (20:4)	LysoPE 20:4	[M–H]–	1.313	500.28
Set -21	Phosphatidylcholine	UCD.Lipid.394	PC 35:4	PC (35:4)	PC(15:0_20:4)	[M+Ac–H]–	4.765	826.56
Set -21	Phosphatidylcholine	UCD.Lipid.398	PC 36:4 B	PC (36:4) B	PC(16:0_20:4)	[M+Ac–H]–	5.074	840.58
Set -21	Phosphatidylcholine	UCD.Lipid.401	PC 37:4	PC (37:4)	PC(17:0_20:4)	[M+Ac–H]–	5.39	854.59
Set -21	Phosphatidylcholine	UCD.Lipid.404	PC 38:4 A	PC (38:4) A	PC(18:0_20:4)	[M+Ac–H]–	5.706	868.61
Set -21	Phosphatidylcholine	UCD.Lipid.407	PC 40:4	PC (40:4)	PC(18:0_22:4)	[M+Ac–H]–	6.172	896.64
Set -22	Phosphatidylethanolamine	UCD.Lipid.168	PE p-34:1	PE (p-34:1) or PE (o-34:2)	PE(16:0_18:1)	[M+H]+	6.16	702.5404
Set -22	Phosphatidylethanolamine	UCD.Lipid.438	PE p-34:2	PE (p-34:2) or PE (o-34:3)	PE(P-16:0_18:2)	[M–H]–	5.64	698.51
Set -22	Phosphatidylethanolamine	UCD.Lipid.440	PE p-36:2	PE (p-36:2) or PE (o-36:3)	PE(P-18:0_18:2)	[M–H]–	6.314	726.54
Set -22	Phosphatidylethanolamine	UCD.Lipid.441	PE p-36:4	PE (p-36:4) or PE (o-36:5)	PE(P-16:0_20:4)	[M–H]–	5.523	722.51
Set -22	Phosphatidylethanolamine	UCD.Lipid.444	PE p-38:3	PE (p-38:3) or PE (o-38:4)		[M–H]–	6.48	752.56
Set -22	Phosphatidylethanolamine	UCD.Lipid.445	PE p-38:4	PE (p-38:4) or PE (o-38:5)	PE(P-18:0_20:4)	[M–H]–	6.189	750.54
Set -22	Phosphatidylethanolamine	UCD.Lipid.446	PE p-38:5	PE (p-38:5) or PE (o-38:6)	PE(P-18:0_20:5)	[M–H]–	5.582	748.53
Set -22	Phosphatidylethanolamine	UCD.Lipid.449	PE p-40:5	PE (p-40:5) or PE (o-40:6)	PE(P-18:0_22:5)	[M–H]–	6.214	776.56
Set -23	Phosphatidylcholine	UCD.Lipid.161	PC p-40:5	PC (p-40:5) or PC (o-40:6)		[M+H]+	5.74	820.6215
Set -23	Phosphatidylcholine	UCD.Lipid.162	PC p-40:6	PC (p-40:6) or PC (o-40:7) A		[M+H]+	5.16	818.6058
Set -23	Phosphatidylcholine	UCD.Lipid.163	PC p-40:6	PC (p-40:6) or PC (o-40:7) B		[M+H]+	5.625	818.6058
Set -23	Phosphatidylcholine	UCD.Lipid.164	PC p-40:7	PC (p-40:7) or PC (o-40:8)		[M+H]+	5.085	816.5902
Set -23	Phosphatidylethanolamine	UCD.Lipid.447	PE p-38:6	PE (p-38:6) or PE (o-38:7)	PE(16:0_22:6)	[M–H]–	5.34	746.51
Set -23	Phosphatidylethanolamine	UCD.Lipid.450	PE p-40:6	PE (p-40:6) or PE (o-40:7)	PE(P-18:0_22:6)	[M–H]–	5.998	774.54
Set -23	Phosphatidylethanolamine	UCD.Lipid.451	PE p-40:7	PE (p-40:7) or PE (o-40:8)	PE(P-18:1_22:6)	[M–H]–	5.407	772.53
Set -24	Phosphatidylethanolamine	UCD.Lipid.166	PE 36:4	PE (36:4)	PE(16:0_20:4)	[M+H]+	5.25	740.5184
Set -24	Phosphatidylcholine	UCD.Lipid.429	PE 34:1	PE (34:1)	PE(16:0_18:1)	[M–H]–	5.831	716.52
Set -24	Phosphatidylethanolamine	UCD.Lipid.430	PE 34:2	PE (34:2)	PE(16:0_18:2)	[M–H]–	5.332	714.51
Set -24	Phosphatidylethanolamine	UCD.Lipid.432	PE 36:2	PE (36:2)	PE(18:0_18:2)	[M–H]–	5.989	742.54
Set -24	Phosphatidylethanolamine	UCD.Lipid.433	PE 36:3	PE (36:3)	PE(18:1_18:2)	[M–H]–	5.399	740.52
Set -24	Phosphatidylethanolamine	UCD.Lipid.435	PE 38:4 B	PE (38:4) B	PE(18:0_20:4)	[M–H]–	5.881	766.54
Set -24	Phosphatidylethanolamine	UCD.Lipid.436	PE 38:6	PE (38:6)	PE(16:0_22:6)	[M–H]–	5.074	762.51
Set -25	Phosphatidylcholine	UCD.Lipid.140	PC 35:3	PC (35:3)		[M+H]+	4.861	770.5694
Set -25	Phosphatidylcholine	UCD.Lipid.141	PC 36:3 B	PC (36:3) B	PC(18:1_18:2)	[M+H]+	5.184	784.5848
Set -25	Phosphatidylcholine	UCD.Lipid.144	PC 37:3	PC (37:3)		[M+H]+	5.425	798.6004
Set -25	Phosphatidylcholine	UCD.Lipid.397	PC 36:3 A	PC (36:3) A	PC(18:1_18:2)	[M+Ac–H]–	5.23	842.59
Set -25	Phosphatidylcholine	UCD.Lipid.402	PC 38:2	PC (38:2)	PC(20:0_18:2)	[M+Ac–H]–	6.389	872.64
Set -25	Phosphatidylcholine	UCD.Lipid.403	PC 38:3	PC (38:3)	PC(18:0_20:3)	[M+Ac–H]–	5.981	870.62

Set-25	Phosphatidylinositol	UCD.Lipid.459	PI 38:3	PI (38:3)	PI(18:0_20:3)	[M–H]–	4.9	887.57
Set-26	Triacylglycerol	UCD.Lipid.235	TG 56:8	TG (56:8)	TG(18:1_18:2_20:5)	[M+Na]+	9.769	925.7256
Set-26	Triacylglycerol	UCD.Lipid.236	TG 56:9	TG (56:9)	TG(18:2_18:2_20:5)	[M+Na]+	9.48	923.7057
Set-26	Triacylglycerol	UCD.Lipid.240	TG 58:10	TG (58:10)	TG(18:2_18:2_22:6)	[M+Na]+	9.396	949.7256
Set-26	Triacylglycerol	UCD.Lipid.245	TG 58:8	TG (58:8)	TG(18:1_18:2_22:5)	[M+NH4]+	10.17	948.8015
Set-26	Triacylglycerol	UCD.Lipid.246	TG 58:9	TG (58:9)	TG(18:2_18:2_22:5)	[M+Na]+	9.794	951.7412
Set-26	Triacylglycerol	UCD.Lipid.249	TG 60:11	TG (60:11)		[M+NH4]+	9.645	970.7859
Set-27	Sphingomyelin	UCD.Lipid.172	SM d42:2	SM (d42:2)	SM(d18:1_24:1)	[M+H]+	6.685	813.6873
Set-27	Sphingomyelin	UCD.Lipid.488	SM d42:2 A	SM (d42:2) A	SM(d18:1_24:1)	[M+Ac–H]–	6.863	871.69
Set-27	Sphingomyelin	UCD.Lipid.489	SM d42:3	SM (d42:3)	SM(d18:2_24:1)	[M+Ac–H]–	6.289	869.68
Set-27	Sphingomyelin	UCD.Lipid.492	SM d44:2	SM (d44:2)		[M+Ac–H]–	7.528	899.72
Set-28	Cholesteroyl ester	UCD.Lipid.11	CE 18:1	CE (18:1)	18:1 Cholesteryl ester	[M+Na]+	10.88	673.5894
Set-28	Cholesteroyl ester	UCD.Lipid.13	CE 18:3	CE (18:3)	18:3 Cholesteryl ester	[M+Na]+	9.985	669.5581
Set-28	Cholesteroyl ester	UCD.Lipid.14	CE 20:3	CE (20:3)	20:3 Cholesteryl ester	[M+NH4]+	10.49	692.634
Set-28	Cholesteroyl ester	UCD.Lipid.15	CE 20:4	CE (20:4)	20:4 Cholesteryl ester	[M+Na]+	10.16	695.5738
